# Discovering the Protective Effects of Quercetin on Aflatoxin B1-Induced Toxicity in Bovine Foetal Hepatocyte-Derived Cells (BFH12)

**DOI:** 10.3390/toxins15090555

**Published:** 2023-09-06

**Authors:** Marianna Pauletto, Mery Giantin, Roberta Tolosi, Irene Bassan, Anisa Bardhi, Andrea Barbarossa, Ludovica Montanucci, Anna Zaghini, Mauro Dacasto

**Affiliations:** 1 Department of Comparative Biomedicine and Food Science, University of Padua, Viale dell’Università 16, I-35020 Legnaro, Italy; mery.giantin@unipd.it (M.G.); roberta.tolosi@unipd.it (R.T.); irene.bassan2@gmail.com (I.B.); mauro.dacasto@unipd.it (M.D.); 2 Department of Veterinary Medical Sciences, Alma Mater Studiorum—University of Bologna, Via Tolara di Sopra 50, Ozzano dell’Emilia, I-40064 Bologna, Italy; anisa.bardhi@unibo.it (A.B.); andrea.barbarossa@unibo.it (A.B.); anna.zaghini@unibo.it (A.Z.); 3 Genomic Medicine Institute, Lerner Research Institute, Cleveland Clinic, 9500 Euclid Avenue, Cleveland, OH 44195, USA; montanl@ccf.org

**Keywords:** aflatoxin B1, quercetin, lipid peroxidation, CYP3A, NQO1, bovine, hepatocyte cell line, transcriptome

## Abstract

Aflatoxin B1 (AFB1) induces lipid peroxidation and mortality in bovine foetal hepatocyte-derived cells (BFH12), with underlying transcriptional perturbations associated mainly with cancer, cellular damage, inflammation, bioactivation, and detoxification pathways. In this cell line, curcumin and resveratrol have proven to be effective in mitigating AFB1-induced toxicity. In this paper, we preliminarily assessed the potential anti-AFB1 activity of a natural polyphenol, quercetin (QUE), in BFH12 cells. To this end, we primarily measured QUE cytotoxicity using a WST-1 reagent. Then, we pre-treated the cells with QUE and exposed them to AFB1. The protective role of QUE was evaluated by measuring cytotoxicity, transcriptional changes (RNA-sequencing), lipid peroxidation (malondialdehyde production), and targeted post-transcriptional modifications (NQO1 and CYP3A enzymatic activity). The results demonstrated that QUE, like curcumin and resveratrol, reduced AFB1-induced cytotoxicity and lipid peroxidation and caused larger transcriptional variations than AFB1 alone. Most of the differentially expressed genes were involved in lipid homeostasis, inflammatory and immune processes, and carcinogenesis. As for enzymatic activities, QUE significantly reverted CYP3A variations induced by AFB1, but not those of NQO1. This study provides new knowledge about key molecular mechanisms involved in QUE-mediated protection against AFB1 toxicity and encourages in vivo studies to assess QUE’s bioavailability and beneficial effects on aflatoxicosis.

## 1. Introduction

Mycotoxins are food and feed contaminants produced as secondary metabolites by a few genera of filamentous fungi, eliciting toxic effects in humans and animals. Estimates by the Food and Agricultural Organization (FAO) indicate that ~25% of global food and feed products are contaminated with mycotoxins, with an estimated economic loss of USD 50 million every year [1,2]. However, the FAO estimates appear to fall short of reality [3]. Global warming and climate change, such as an increase in temperature (2°–5°) and CO_2_ concentration (twice or thrice the expected value) as well as drought episodes, have been predicted to affect the occurrence of mycotoxins in the years to come [4].

Mycotoxins that would benefit most from climate change are aflatoxins, a class of natural mycotoxins produced by *Aspergillus flavus* and *A. fumigatus* [1,5]. According to their fluorescence, aflatoxins are broadly classified into four classes: B1, B2, G1, and G2 [6]. Aflatoxins usually affect crops from tropical and/or subtropical areas and are commonly found in mouldy corn, soybeans, rice, sorghum, dried figs, spices, hazelnuts, peanuts, and other grain and oil crops [5,7,8,9]. It is believed that about 4.5 billion people worldwide are at risk of excessive exposure to aflatoxins via contaminated food [7], but until a few years ago, these mycotoxins were not considered a matter of concern in Europe. However, due to global climate change, the presence of fungal strains endemic in tropical or subtropical climate zones has increased in temperate ones. Therefore, the risk of food and feed contamination and, consequently, of human and animal exposure to aflatoxins is expected to become higher than in the past [1,5,10].

Aflatoxin B1 is the primary aflatoxin responsible for food and feed contamination [2], and it is by far the most toxic aflatoxin [2,11]. It exhibits pleiotropic effects in several human and animal tissues; it is immunotoxic, mutagenic, carcinogenic, and teratogenic. The liver is the main target organ of aflatoxin B1. Exposure to aflatoxin B1 has been linked to hepatic carcinogenesis in humans, poultry (e.g., turkey), fish (e.g., trout), and rodents [12,13,14,15]. In particular, epidemiological surveys suggest that dietary aflatoxin B1 exposure contributes to the higher incidence of hepatocellular carcinoma (HCC) in Asia and sub-Saharan Africa [16,17,18]. Additional risk factors are likely to increase the aflatoxin B1-dependent occurrence of HCC, such as hepatitis virus B (HVB) infection [16,19]. Based on these different lines of epidemiological evidence, in 2012, the International Agency for Research on Cancer (IARC) classified aflatoxin B1 and the other aflatoxins, including B1’s major metabolite aflatoxin M1, as carcinogenic to humans (Group I) [10,20].

Aflatoxin B1 is not carcinogenic per se; it is a pro-carcinogen, as it undergoes a bioactivation reaction in the human liver, resulting in the formation of the ultimate carcinogen aflatoxin B1-8,9-epoxide and a number of hydroxylated derivatives [10,11,12]. It is worth mentioning that the cytochromes P450 1A1 and 3A4 (CYP1A1 and CYP3A4, respectively) are phase I drug-metabolizing enzymes playing a critical role in aflatoxin B1 metabolism in human, duck, turkey, and cattle livers [11,21]. Apart from aflatoxin B1 epoxide, the most toxic and carcinogenic aflatoxin derivative is aflatoxin M1. This hydroxylated derivative, together with aflatoxin B1 and variable amounts of aflatoxicol, can be found in the milk and cheese from mycotoxin-exposed dairy cows. Of the farming species, cattle are relatively more resistant to aflatoxins because of the capability of rumen microbiota to convert aflatoxin B1 into less toxic or nontoxic derivatives [22,23]. Nevertheless, dairy milk contamination with aflatoxins remains a serious food safety concern, as it might pose severe health risks to humans consuming dairy food products in general and to susceptible population groups [10,24,25]. To cope with this health concern, approaches focused primarily on limiting aflatoxin B1 absorption and preventing aflatoxin excretion in dairy milk have been adopted, but they are only partially successful [25].

Promising strategies reducing the impact of aflatoxins in cattle farming are those that make use of phytochemical derivatives, such as polyphenols, with strong antioxidant activity [8]. Indeed, besides the aforementioned toxic effects, aflatoxin B1-dependent hepatotoxicity is closely related to the mycotoxin’s capability to generate reactive oxygen species (ROS), resulting in cellular oxidative stress and its well-known consequences (e.g., oxidative DNA damage and membrane lipid peroxidation) [9,16,26]. Overall, these botanical extracts can reduce oxidative damage in the body by increasing the activity of antioxidant enzymes and scavenging free radicals [27]. Moreover, natural antioxidants can take advantage of more pathways and molecular targets. Finally, they are less toxic and have fewer side effects [9]. Therefore, some of these plant-based compounds are increasingly used to prevent aflatoxin B1 formation in toxigenic moulds and to detoxify aflatoxin B1-contaminated food and feed without any deleterious effects on their nutritional value [8,28]. Curcumin, resveratrol, oxidized tea phenolics, and flavonoids such as quercetin are among the most studied anti-aflatoxin polyphenols [8]. To assess the effectiveness and feasibility of these dietary polyphenols in preventing or mitigating aflatoxin B1 contamination of dairy milk, conducting mechanism-based studies in bovine target tissues in vitro and in vivo is a fundamental starting point [9,29].

The aim of the present study was to assess the protective role of quercetin (QUE) in a bovine foetal hepatocyte-derived cell line (BFH12) exposed to aflatoxin B1. To this end, we measured the effects triggered by QUE on aflatoxin B1-induced cytotoxicity and related transcriptional changes (RNA-sequencing). Confirmatory studies on protein expression and biological activity were also performed. This is part of a series of in vitro and in vivo studies aimed at measuring the biological effects of polyphenols on the toxicity of aflatoxin B1 (AFB1) and its derivatives in dairy cows [24,29,30,31,32].

## 2. Results

### 2.1. Quercetin’s Half-Maximal Inhibitory Concentration and Ability to Counteract AFB1 Cytotoxicity

Bovine cells were incubated with increasing concentrations of QUE to build up a dose–response curve (Figure S1). The fitting of the data was not excellent (R squared = 0.68), but some sigmoidal trend was observed, thus allowing for the estimation of the QUE half-maximal inhibitory concentration (IC_50_) at 64 h, which turned out to be 59.97 μM.

**Figure 1:**
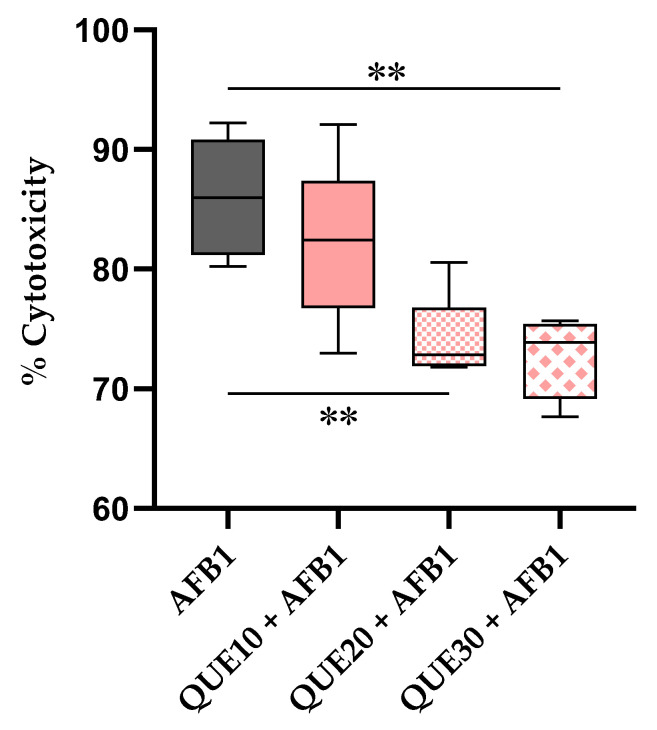
Effect of quercetin on AFB1 cytotoxicity. The box and whiskers plot reports the viability of bovine foetal hepatocyte (BFH12) cells pre-treated (16 h) with increasing QUE concentrations (10, 20, and 30 μM) and exposed (48 h) to a combination of AFB1 3.6 μM and QUE (at the same concentrations above mentioned). **: *p* ≤ 0.01 (ANOVA one-way and Dunnett’s multicomparisons tests; the mean of each condition was compared with that of AFB1). Graphs were obtained using GraphPad prism software. AFB1 = aflatoxin B1; QUE = quercetin.

Afterwards, we assayed the protective role of QUE against AFB1 cytotoxicity. At the tested sub-cytotoxic concentrations, QUE reduced the AFB1 cytotoxicity in a dose-dependent manner. The decrease was statistically significant (*p* ≤ 0.01) with 20 and 30 μM QUE, and the highest QUE concentration reduced the median AFB1 cytotoxicity by 13.39% (from 86.00% to 72.61%; Figure 1).

### 2.2. Effect of QUE on AFB1 Biotransformation in BFH12 Cells

**Figure 2:**
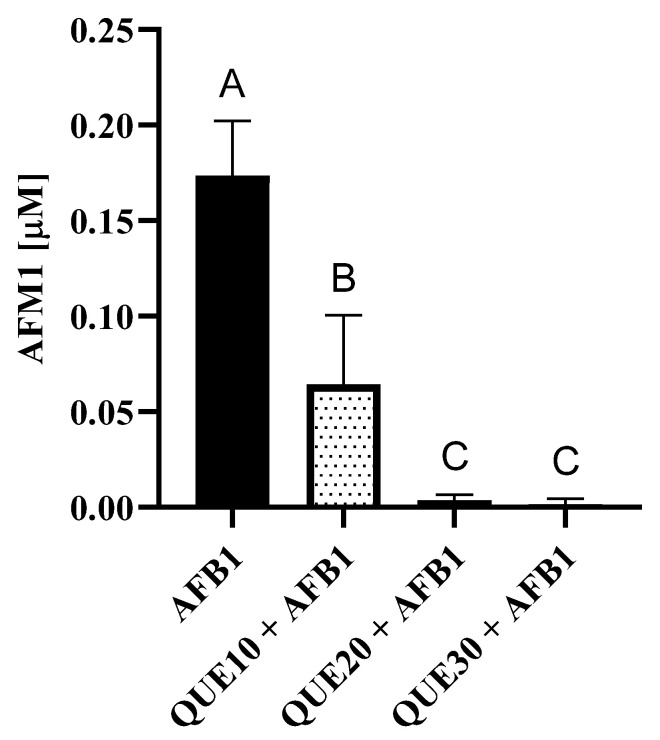
Effect of quercetin on AFB1 biotransformation. Bars represent the amount (μM) of aflatoxin M1 (AFM1) detected in the cellular medium after 48 h of exposure to 3.6 μM AFB1 alone or in combination with increasing QUE concentrations (10, 20, and 30 μM). Data are expressed as mean concentrations ± standard deviations of four independent cell culture experiments. Different letters above error bars indicate significant differences (*p* ≤ 0.05) among groups (Tukey’s post hoc test). Graphs were obtained using GraphPad prism software.

Quercetin significantly decreased the amount of aflatoxin M1 (AFM1) in the cellular medium in a dose-dependent manner. In particular, the AFM1 concentration dropped from 57.00 ng/mL (i.e., 0.174 μM) to 0.5 ng/mL (0.002 μM; Figure 2). As for AFL, cells exposed to AFB1 + 10 μM QUE showed significantly higher amounts of this AFB1 derivative, i.e., from 85.5 ng/mL (0.272 μM) to 142.5 ng/mL (0.453 μM). However, at medium and maximum QUE concentrations, the amount of aflatoxicol (AFL) was similar to the amount detected in the presence of AFB1 alone (Figure S2a).

Detectable amounts of AFM1 and AFL have never been found in cell pellets; however, the incubation with increasing QUE concentrations led to an increase in AFB1 concentration, i.e., from 9.79 ng/mL (0.031 μM) to 47.50 ng/mL (0.152 μM), but these variations were not statistically significant (Figure S2b).

### 2.3. Effects of QUE, Alone or in the Presence of AFB1, on Selected AFB1 Target Genes (qPCR)

#### 2.3.1. Drug Metabolizing Enzymes (DMEs)

**Figure 3:**
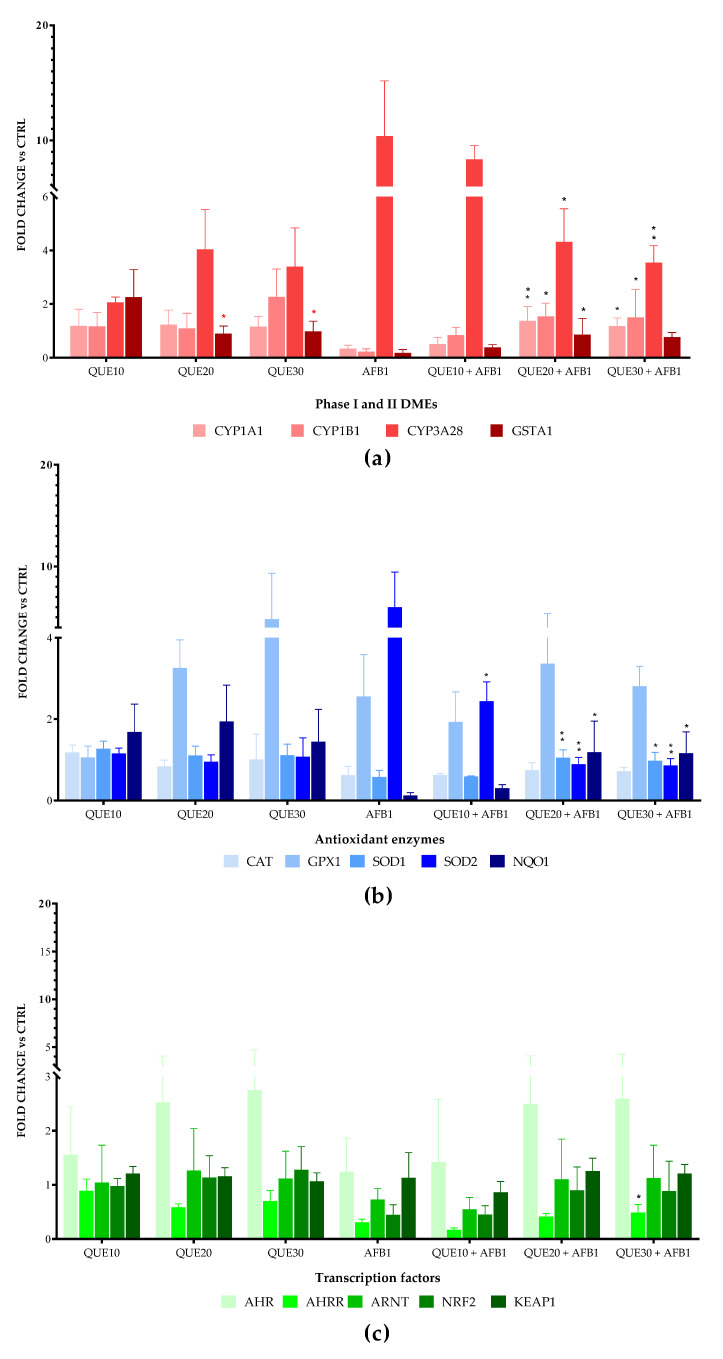
Quercetin-induced changes in mRNA levels of oxidative (phase I) and conjugative (II) drug metabolizing enzymes (DMEs, (**a**)), antioxidant enzymes (AOEs, (**b**)) and related transcription factors (TF, (**c**)). The one-way ANOVA, followed by a Dunnett’s multi-comparisons test, was carried out to compare the gene expression level of BFH12 cells exposed to increasing concentrations of QUE (10, 20, and 30 μM; the lowest dose was used as control in the comparisons); significant variations were identified by red asterisks. The same statistical approach was adopted to separately investigate the effects of QUE pre-treatment in the presence of AFB1 3.6 μM: all the co-treatment conditions were compared to the AFB1 condition; significant variations were identified by black asterisks. Data (means ± standard deviations) are expressed as n-fold changes normalized to ΔΔCt mean value of control (i.e., polychlorinated biphenyls 126, PCB126) to whom an arbitrary value of 1 was assigned. *: *p* ≤ 0.05; **: *p* ≤ 0.01 (ANOVA one-way and Dunnett’s multicomparisons test). Graphs were obtained using GraphPad prism software. AHR = aryl hydrocarbon receptor; AHRR = aryl hydrocarbon receptor repressor; ARNT = aryl hydrocarbon receptor nuclear translocator; CAT = catalase; CTRL = control; CYP1A1 = cytochrome P450 1A1; CYP1B1 = cytochrome P450 1B1; CYP3A28 = cytochrome P450 3A28; DMEs = drug metabolizing enzymes; GPX = glutathione peroxidase I; GSTA1 = glutathione S-transferase A1; KEAP = kelch like ECH associated protein 1; NQO1 = NAD(H):quinone oxidoreductase 1; NRF2 = nuclear factor erythroid 2-related factor 2; SOD1 = superoxide dismutase 1; SOD2 = superoxide dismutase 2.

The incubation with increasing amounts of QUE downregulated the expression of glutathione S-transferase A1 (GSTA1) (Figure 3a).

In cells pre-treated with QUE and then exposed to AFB1, substantial transcriptional changes were recorded at the highest QUE concentrations (20 and 30 μM). An overall significant gene upregulation was observed for CYP1A1, CYP1B1, and GSTA1. The most significant variations were observed for the expression of CYP3A28, the bovine orthologue of human CYP3A4 [33]. A significant and dose-dependent fold change decrease was observed with 20 and 30 μM QUE (Figure 3a).

#### 2.3.2. Antioxidant Enzymes (AOEs)

The pre-incubation with the chosen QUE concentrations did not result in statistically significant variations in mRNA levels of the most important AOEs (Figure 3b). 

In cells co-exposed to AFB1 and QUE, as compared to cells exposed to AFB1 alone, significant transcriptional changes were observed only with 20 and 30 μM QUE: superoxide dismutase 1 (SOD1) and quinone oxidoreductase 1 (NQO1) genes were upregulated, while SOD2 was significantly and dose-dependently downregulated (Figure 3b).

#### 2.3.3. Transcription Factors (TFs)

Genes coding for DMEs and AOEs recognize a number of TFs involved in their transcriptional mechanisms of gene regulation and play an important role in AFB1 toxicity [34,35]. No statistically significant variations in the mRNA of target TFs were observed in cells pre-incubated with either QUE alone or QUE in combination with AFB1, with the sole exception of the aryl hydrocarbon receptor repressor (AHRR), showing a slight but significant induction in cells pre-treated with 30 μM QUE (Figure 3c).

### 2.4. Differential Expression Analysis

To obtain clear transcriptional results, RNA-sequencing (RNA-seq) investigations were carried out only in cells treated with the highest QUE concentration (30 μM). 

A total of 296,495,475 raw reads were sequenced and deposited in GeneBank under BioProject accession PRJNA627332. These reads were subjected to quality control measures, and after trimming and rRNA removal, 24.5 million reads per sample were retained on average. Approximately 99% of the obtained reads were mapped to the *B. taurus* reference genome (Table S1). The MDS plot in Figure S3 shows an unsupervised clustering of the samples. The first dimension (x-axis) clearly separates samples based on the experimental group. Major differences were observed between cells exposed to AFB1 (bottom right) and control cells (bottom left). Both the first and the second dimension (y-axis) separate the transcriptional profiles observed in cells exposed to QUE (alone or in combination with AFB1), thus forming a unique cluster in the plot (top centre). Biological variability within experimental groups is low, as demonstrated by the coherent clusters formed by the replicas. 

**Figure 4:**
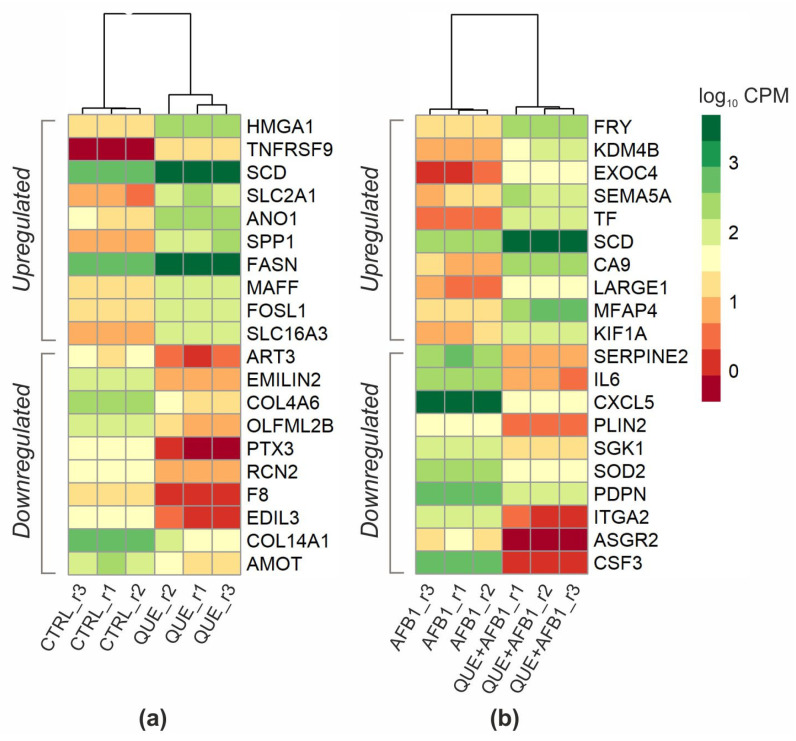
Gene expression levels of differentially expressed genes. Heatmap of top 10 genes up- and downregulated by QUE (vs. CTRL) (**a**) and QUE + AFB1 co-treatment (vs. AFB1) (**b**). Heatmaps were constructed in R environment using the pheatmap package and using the normalized log10CPM (Counts Per Million) as input. Biological replicates are represented by r1, r2, and r3 suffixes.

When comparing the transcriptional profiles of cells exposed to QUE and those of control cells, a total of 1028 differentially expressed genes (DEGs) were found: 487 were upregulated, and 541 were downregulated (Figure S4). The edgeR output of the DE analysis conducted in this study is reported in Table S2. Among the top 10 upregulated genes (Figure 4a), we found QUE transporter solute carrier family 2 member 1 (SLC2A1, also known as GLUT1), with a log_2_ fold change (lfc) of 4.72; players in inflammatory processes, cell proliferation, and survival, such as tumour necrosis factor superfamily member 9 (TNFRSF9, lfc = 5.13); high-mobility group at-hook 1 (HMGA1, lfc = 3.34); secreted phosphoprotein 1 (*SPP1*, also known as osteopontin; lfc = 3.82); and the MAP BZIP transcription factor (MAFF, lfc = 2.37), a gene coding for a small Maf protein and involved in the cellular stress response. Additionally, QUE significantly increased the expression of two enzymes involved in lipid metabolism, i.e., stearoyl-CoA desaturase (SCD, lfc = 3.22) and fatty acid synthase (FASN, lfc = 2.89). Among the top 10 downregulated genes, we found a gene coding for an acute phase protein, pentraxin 3 (PTX3, lfc = −5.42), and two genes implicated in carcinogenic processes, i.e., olfactomedin-like 2B (OLFML2B, lfc = −3.22) and elastin microfibril interfacer 2 (EMILIN2, lfc = −3.33).

The enrichment analysis, carried out following a more comprehensive analysis of DEGs, resulted in the significant enrichment of 19 Biological Processes (BPs) and 17 Kyoto Encyclopaedia of Genes and Genomes (KEGGs) (Table S3, Figure S5). Among the significant BPs, there was a “steroid metabolic process” (gene count = 16), represented by genes involved in the metabolism of steroids and other substrates (e.g., fatty acids, prostaglandins, and xenobiotics), such as hydroxysteroid dehydrogenases HSD 3B1 (lfc = 2.54) and HSD17B12 (lfc = 1.95), and key transcription factors in the regulation of cholesterol metabolism, such as SCARB1. Several genes belonging to this enriched BP are involved in regulating cholesterol biosynthesis, including sterol regulatory element-binding transcription factor 2 (SREBF2; lfc = 1.56), HMG-CoA reductase (HMGCR; lfc = 2.62), low-density lipoprotein receptor (LDLR; lfc = 3.24), and 7-dehydrocholesterol reductase (DHCR7; lfc = 2.39). 

An additional significantly enriched BP was “response to external stimulus” (gene count = 47). This BP was represented by both up- and downregulated genes. With regard to genes induced by QUE, we highlight prostaglandin-endoperoxide synthase 2 (PTGS2; lfc = 2.82), a member of the Forkhead box (FOXA3; lfc = 2.19), glutathione peroxidase 1 (GPX1; lfc = 1.51), and adrenoceptor beta 2 (ADRB2; lfc = 2.77). With regard to genes downregulated by QUE, we found complement factors C2 (lfc = −2.41) and H (CFH, lfc = −4.78), C-X-C motif chemokine ligands 9, 10, and 11 (lfc = −4.57, −4.15, −5.16, respectively), fatty acid-binding protein 4 (FABP4; lfc = −4.37), and the CD36 molecule (lfc = −2.15).

Like “response to external stimulus”, the BP term “response to wounding” (gene count = 13) was also significantly enriched. This BP was represented by some DEGs already listed in the “response to stimulus” BP and some additional genes, such as von Willebrand factor (VWF; lfc = 2.73) and claudin 1 (CLDN1; lfc = 1.88).

Finally, an interesting enriched BP was “inflammatory response” (gene count = 18), with several genes shared with the “response to external stimulus” BP and some additional ones, such as prostaglandin-endoperoxide synthase 1 (PTGS1; lfc = −2.30).

Enriched KEGGs provided further insights into the molecular changes triggered by QUE. Interestingly, some KEGGs related to immunity were significantly enriched, such as “neutrophil extracellular trap formation” (gene count = 26) and “complement and coagulation cascades” (gene count = 10). With regard to complement factors, while C2 and CFH were significantly downregulated, a gene encoding a complement receptor (C3AR1) was upregulated, with an lfc of 2.94. 

Notable enriched KEGGs were “PPAR signalling pathway” (gene count = 13) and “drug metabolism—cytochrome P450” (gene count = 9). In the PPAR pathway, significant genes code for fatty acid-binding proteins 3, 5, and 4 (lcf = 3.50, −4.33, −4.37, respectively) and enzymes were involved in the biosynthesis and degradation of cellular lipid fatty acids. The “P450-mediated drug metabolism” KEGG was mostly represented by key antioxidant enzymes and glutathione S-transferases, including GSTA3 (lfc = 8.32), GSTA2 (lfc = 3.01), GSTM1 (lfc= 2.11), GSTK1 (lfc = −1.61), and GSTT4 (lfc = −1.94).

Gene expression profiles of cells co-treated with QUE and AFB1 were compared to those of cells treated with AFB1 alone. This comparison allowed for the identification of 1890 DEGs (Table S2). Most of the significant genes, 1275, were upregulated, while 615 genes were downregulated (Figure S4). The expression levels of the top 10 up- and downregulated genes are reported in a heatmap (Figure 4b). Noteworthily, among the top 10 upregulated DEGs, we found genes related to carcinogenesis, such as lysine demethylase 4B (KDM4B, lfc = 3.41), stearoyl-CoA desaturase (SCD, lfc = 3.75), and carbonic anhydrase 9 (CA9, lfc = 4.60). Top upregulated genes also included FRY microtubule-binding protein (FRY, lfc = 3.24), semaphorin 5A (SEMA5A, lfc = 3.56), and transferrin (TF, lfc = 5.13). Among the top downregulated DEGs, we found genes implicated in inflammatory processes, such as chemokine (C-X-C motif) ligand 5 (CXCL5, lfc = −6.16), interleukin 6 (IL6, lfc = −5.72), colony-stimulating factor 3 (CSF3, lfc = −8.19), and podoplanin (PDPN, lfc = −2.73), which also play an important role in liver fibrosis and cancer. A key role in the liver is also played by another gene, perilipin 2 (PLIN2; lfc = −3.63), a marker of steatosis, which is downregulated here. Like PDPN, serum/glucocorticoid-regulated kinase 1 (SGK1, lfc = −2.86), listed among the top 10 genes downregulated by QUE pre-treatment, is known to play a crucial role in tumourigenesis and cancer progression. A further gene whose expression was strongly reduced by QUE pre-treatment was the key antioxidant enzyme SOD2 (lfc = −2.87), which is consistent with the qPCR data (Figure 3b). As observed in cells exposed to only QUE, microsomal glutathione S-transferase 1 (MGST1), GSTA2, GSTM1, and GSTK1 were significantly regulated, with lfc values of 3.20, 4.96, 3.01, and −1.40, respectively.

The enrichment analysis conducted on the list of 1890 DEGs resulted in the significant enrichment of 6 BPs and 10 KEGGs (Table S3, Figure S6). Overall, some terms modulated by the co-treatment QUE + AFB1 were also significantly affected by QUE alone, e.g., “response to external stimulus”, “steroid biosynthesis”, and “neutrophil extracellular trap formation”, yet the DEGs involved were not completely the same. As far as the DEGs representing the enriched BP “response to external stimulus” (gene count = 73) are concerned, we found many genes playing a role in mediating inflammatory processes, such as prostaglandin I2 synthase (PTGIS, lfc = 3.09), CXCL5 (lfc = −6.16), C-C motif chemokine ligand 2 (CCL2; lfc = −3.82), CCL20 (lfc = −4.91), and high-mobility group box 1 (HMGB1; lfc = −1.30), in addition to genes regulating apoptosis, such as BCL2 interacting protein 3 like (BNIP3L; lfc = 1.79) and interferon alpha inducible protein 6 (IFI6; lfc = 2.01).

In the analysis of the effects of QUE alone, the pathway of steroid metabolism appeared to be significantly affected by the co-treatment QUE + AFB1, as demonstrated by the enrichment of the BP term “cholesterol biosynthetic process” (gene count = 9) and the KEGG pathway “steroid biosynthesis” (gene count = 11). Specifically, the co-treatment seems to mostly upregulate genes of the cholesterol biosynthesis, rather than steroids in general, such as LPCAT3 (lfc = 1.85), SREBF2 (lfc = 2.49), lanosterol synthase (LSS; lfc = 2.41), DHCR7 (lfc = 3.02), and HMGCR (lfc = 2.86).

Innate and inflammatory host defences were significantly enriched, as demonstrated by the KEGG pathways “rheumatoid arthritis” (gene count = 19), “systemic lupus erythematosus” (gene count = 18), “neutrophil extracellular trap formation” (gene count = 28), and “cytokine-cytokine receptor interaction” (gene count = 31). In these pathways, we found many mediators of inflammation, such as intercellular adhesion molecule-1 (ICAM1; lfc = −2.62), interleukin 1-alpha (IL1A; lfc = −3.19), interleukin 6 signal transducer (IL6ST; lfc = −1.75), interleukin 7 receptor (IL7R; lfc = −2.75), CXCL3 (lfc = −3.89), LIF interleukin 6 family cytokine (LIF; lfc = −2.73), interleukin 1 receptor antagonist (IL1RN; lfc = 2.28), CD40 (lfc = −2.35), colony-stimulating factor 2 (CSF2; lfc = −2.79), and C2 (lfc = −2.73). 

A further significantly enriched KEGG was “ABC-transporters” (gene count = 12), with both up- and downregulated genes. Notably, the mRNA expression of ABCG1, ABCA2, and ABCD1 was induced by QUE + AFB1, with lfc values of 2.81, 2.37, and 1.35, while the mRNA expression of ABCB1 and ABCC4 was downregulated, with lfc values of −1.55 and −2.07, respectively. 

**Figure 5:**
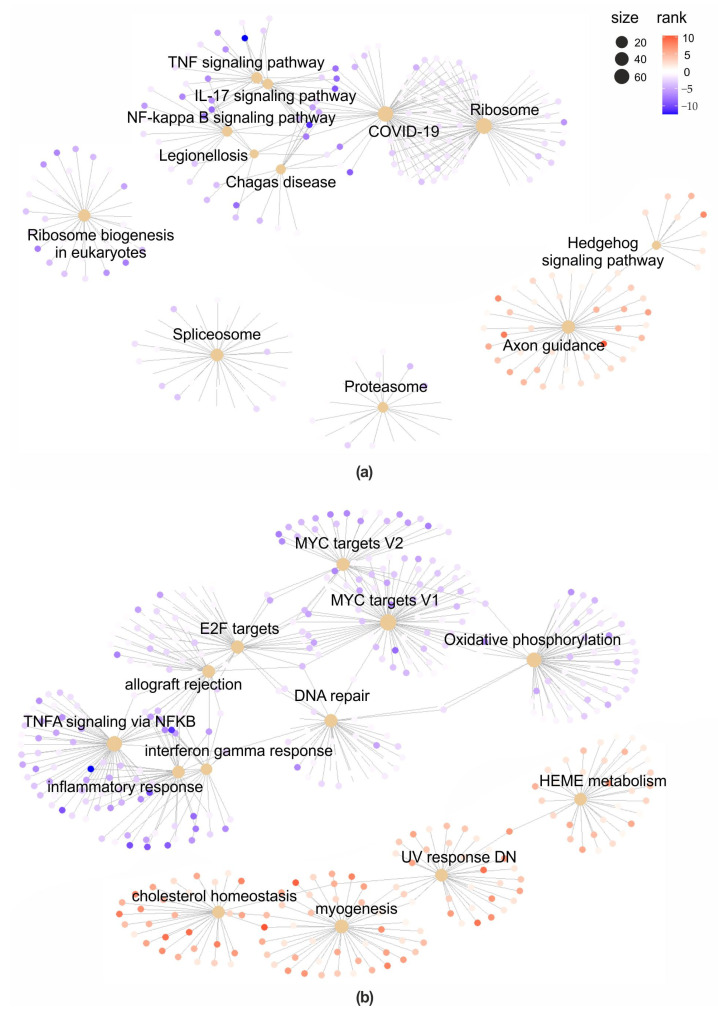
Gene Set Enrichment Analysis (QUE + AFB1 vs. AFB1). Gene-concept network of the significantly enriched KEGG pathways (**a**) and Hallmark gene sets (**b**). Node size reflects the gene set size (i.e., number of genes). The dots colour (from blue to red) indicates genes ranking (highly down- or upregulated).

Finally, gene set enrichment analysis (GSEA) highlighted a suppression of several pathways involved in inflammation in the co-treated cells, compared to cells treated with AFB1 alone (Figure 5a,b, Table S4). Some examples are represented by the KEGG pathways “IL-17 signalling pathway” (normalized enrichment score [NES] = −1.99), “NF-kappa B signalling pathway” (NES = −1.73), and “TNF signalling pathway” (NES = −1.63), as well as the hallmark pathways “TNFA signalling via NFKB” (NES = −1.92), “inflammatory response” (NES = −1.58), and “interferon gamma response” (NES = −1.35). Notably, some pathways related to carcinogenesis were also significantly suppressed in the presence of QUE, such as “MYC targets V1” (NES = −2.33), “MYC targets V2” (NES = −2.07), and “DNA repair” (NES = −1.66).

**Figure 6:**
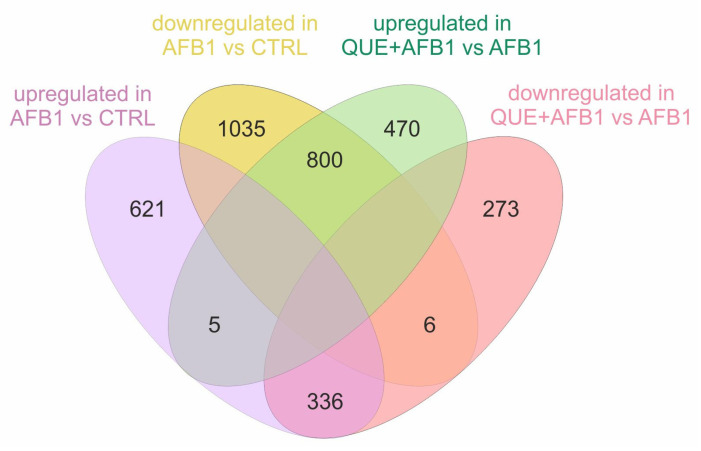
Shared differentially expressed genes. Venn diagram of shared DEGs in the comparison between AFB1 vs. CTRL and QUE + AFB1 vs. AFB1.

Although the analysis of the transcriptional effects of AFB1 is beyond the scope of the present study and has already been performed in a previous experiment [32], we compared the DEGs from QUE + AFB1 vs. AFB1 and those from AFB1 vs. control (CTRL) (i.e., 2803 genes). We observed that 60% of the total number of DEGs in the comparison QUE + AFB1 vs. AFB1 (i.e., 1136 out of 1890) exhibited a transcriptional regulation opposite to that in the comparison AFB1 vs. CTRL (Figure 6). Specifically, the transcriptional level of 336 genes was increased by AFB1 when compared to CTRL, but it was decreased by the co-treatment QUE + AFB1 when compared to AFB1 alone. Likewise, the expression of 800 genes decreased in response to AFB1 but increased in the condition QUE + AFB1.

**Figure 7:**
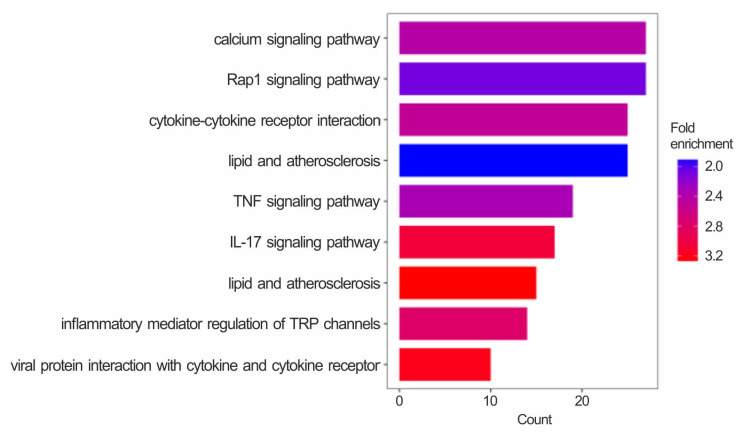
Enrichment analysis of the shared DEGs. Bar plots report the enriched KEGG pathways (FDR ≤ 0.05) in the list of shared DEGs in the pair-wise comparisons QUE + AFB1 vs. AFB1 and AFB1 vs. CTRL. Count = number of DEGs in each enriched pathway. The colour gradient reflects the fold enrichment of each term.

The KEGG functional enrichment of these 1136 genes (Figure 7) revealed some terms identical or similar to those from the functional analyses conducted on the gene expression differences between cells co-treated with QUE + AFB1 and those treated with AFB1 alone (Figure 5 and Figure S4, Tables S3 and S4). In particular, these terms were mainly related to immunity and inflammation (i.e., IL-17 signalling, cytokine-cytokine interactions, TNF signalling, and lipids and atherosclerosis) and calcium signalling.

Finally, the RNA-seq results were compared to those obtained using qPCR (Figure 3) in order to check the consistency between the two methods. The two approaches were mostly in agreement (Figure S7).

### 2.5. Effect of QUE on Possible AFB1-Dependent Oxidative Stress

**Figure 8:**
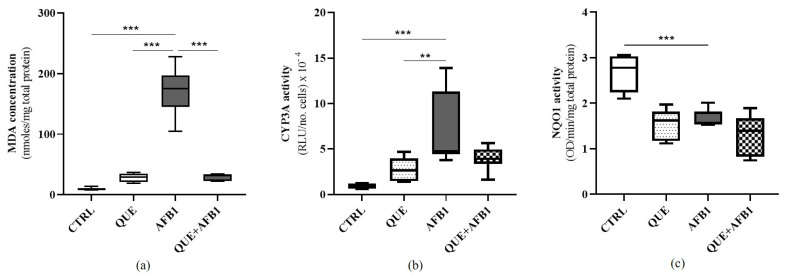
Effect of quercetin on AFB1-dependent oxidative stress, cytochrome P450 3A (CYP3A) and NQO1 activities. The box and whiskers plots report the amount of malondialdehyde (MDA, (**a**)) and the enzyme activity of CYP3A (**b**) and NQO1 (**c**) measured in the cellular medium either from control than in QUE (30 μM), AFB1 (3.6 μM), and QUE + AFB1 (30 μM and 3.6 μM, respectively)-exposed BFH12 cells. The statistical comparisons were established between the median values reported in cells exposed to AFB1 and those observed in all other experimental conditions. **: *p* ≤ 0.01; ***: *p* ≤ 0.001 (ANOVA one-way and Dunnett’s multicomparisons tests). Graphs were obtained using GraphPad prism software. CYP3A = cytochrome P450 3A; MDA = malondialdehyde; OD = optical density; RLU = relative light units.

The generation of ROS, oxidative stress, and lipid peroxidation are among the mechanisms of AFB1-induced toxicity [2,9,12]. To assess the counteracting effect of QUE on such a sequel of toxic events, we measured the amount of malondialdehyde (MDA), a known marker of lipid peroxidation. AFB1 significantly increased (*p* ≤ 0.001) the amount of MDA compared to control cells (Figure 8a). However, QUE did not show a significant increase in this parameter. Importantly, the co-incubation of BFH12 cells with QUE and AFB1 revealed a significant decrease (*p* ≤ 0.001) in MDA amount compared to cells exposed to AFB1 alone, thus confirming the flavonoid’s potential to counteract AFB1-dependent oxidative damage.

### 2.6. Effect of QUE on Target Enzyme Activity (CYP3A and NQO1)

According to the present gene expression results and similarly to previously published results [29,31], we measured the activity of two enzymes known to be involved in AFB1 bioactivation and the antioxidant response(i.e., CYP3A and NQO1, respectively) [11,36].

With regard to CYP3A catalytic activity, cells exposed to AFB1 showed a statistically significant increase in CYP3A activity when compared to that of control and QUE pre-incubated cells (*p* ≤ 0.001 and *p* ≤ 0.01, respectively). By contrast, the combined exposure to AFB1 and QUE considerably reduced the CYP3A activity, bringing it back to levels slightly above than that of the control (Figure 8b).

An overall decrease in NQO1 activity (and, consequently, in cellular antioxidant response) was observed in BFH12 cells exposed to QUE, AFB1, and their combinations. Nevertheless, such a decrease was statistically significant only in AFB1-exposed cells (*p* ≤ 0.001; Figure 8c). This is in opposition to what we observed at the transcriptional level. Indeed, the qPCR revealed a slightly significant increase in NQO1 activity at the highest QUE concentrations (20 and 30 μM + AFB1), compared to AFB1 alone (Figure 3b). Likewise, the RNA-seq analysis (QUE + AFB1 vs. AFB1) identified this gene as upregulated (Table S2).

## 3. Discussion

For many years, the antioxidant properties of polyphenols were assumed to result merely from their capacity to donate electrons or chelate transition metals. However, recent studies have shown that they might have multiple modes of action, interfering with several cell signalling pathways [37,38,39,40,41]. Although several studies have been conducted on polyphenols’ positive effects in human medicine [42,43] and animal farming [44,45,46,47], only marginal attention has been paid to the underlying molecular mechanisms, which are not yet fully understood.

In cattle, the in vitro benefits of QUE have been evaluated very recently. A potential application of QUE in treating bovine viral diarrhoea virus infection has been explored in Madin–Darby bovine kidney cells (MDBK) [48]. Likewise, QUE’s anti-inflammatory potential has been demonstrated in bovine intestinal epithelial cells (BIECs) [49] and mammary epithelial cells (BMECs) [50]. The above-mentioned studies provided important molecular results to disentangle QUE’s mechanism of action in cattle, thus increasing the fundamental knowledge that is necessary to introduce QUE-supplemented feed in farming practice. However, to our knowledge, sequencing technologies querying the entire genome, such as RNA-seq, have never been employed in cattle, neither in vitro nor in vivo. 

Therefore, in the present study, we primarily assessed the toxicity and the transcriptional effects of QUE in a selected bovine hepatic cellular model, BFH12. Then, we proved QUE’s ability to mitigate AFB1 toxicity in vitro and demonstrated the underlying molecular pathways.

### 3.1. Cytotoxicity of Quercetin

The effects of QUE have previously been studied in different cell lines, and the published IC_50_ values were consistent with the results obtained in the present study. After 48 h of exposure, QUE’s IC_50_ was approximately 50 µM in several in vitro models, such as the human breast cancer cell line MCF-7 [51], the human leukaemia cell lines K562 and CEM [41], and the bovine mammary epithelial cell line BME-UV1 [30]. The IC_50_ of QUE that we observed in this study is consistent with the one estimated after 48 h in the human hepatoma cell line HepG2, i.e., 107 µM [52]. Higher IC_50_ values were found in prostate cancer (PC3) and lung cancer (A549) cell lines, which are clearly less sensitive to QUE [51]. If QUE’s IC_50_ is compared with the IC_50_ values obtained in the same cell line for other well-known polyphenolic compounds [29,31], then QUE is less toxic than curcuminoids but as toxic as resveratrol.

The positive impact of this natural flavonoid in cattle was clearly demonstrated by its ability to significantly decrease AFB1-induced cytotoxicity. Notably, compared to resveratrol [31], QUE appeared to be three times less effective in counteracting AFB1-mediated cytotoxicity; this is particularly relevant considering that these two polyphenols have similar IC_50_ values in BFH12 cells. Conversely, in BME-UV1, QUE exhibited a higher protective ability against AFB1 cytotoxicity than resveratrol [30]. We might hypothesize that the efficacy of different polyphenols varies depending on the target tissues, suggesting overall better outcomes when these natural extracts are used in combination, as recently shown in rat models of colon carcinoma [53].

### 3.2. Biotransformation of AFB1

Aflatoxin M1, one of the most toxic AFB1 derivatives [54], appeared to be inversely correlated with QUE concentration, as previously observed for curcuminoids and resveratrol [29,31]. Most probably, QUE reduces AFM1 production by targeting the major enzymes involved in AFM1 hepatic formation. As an example, CYP3A mRNA expression and catalytic activity were highly enhanced by AFB1 alone, but this increase was reversed via the pre-treatment with QUE, even though at the catalytic activity level, this effect was only close to the threshold of significance. To explain these results, we hypothesize that QUE might modulate the synthesis and/or activity of CYP3A. Nevertheless, to clarify the possible interactions among QUE, AFB1, and CYP3A, target studies are clearly needed. In opposition to what we reported for resveratrol [31], the amount of highly toxic AFL did not increase with flavonoid concentration. It has always been thought that AFL production is not an efficient detoxification reaction because AFL is carcinogenic and a repository for AFB1 [14]. However, AFL production has recently been associated with minor sensitivity to AFB1 by reducing the amount of AFB1 available for bioactivation [55]. Therefore, we can postulate that QUE’s failure to shift AFB1 metabolism towards AFL is one of the mechanisms that make this flavonoid not as effective as resveratrol in reducing AFB1-induced toxicity.

### 3.3. Molecular Effect of QUE Underlying Its Potential Role as an Anti-AFB1

In a previously published study, AFB1 was found to significantly affect the transcriptome of BFH12 cells, eliciting carcinogenesis, cellular damage and apoptosis, inflammation, bioactivation, and detoxification pathways [32]. Here, we demonstrated that a pre-treatment with QUE mitigated AFB1-induced cytotoxicity and profoundly modified the cell line’s transcriptional response to AFB1 exposure. The transcriptional modifications induced by QUE, conferring moderate protection against AFB1 toxicity, were mainly related to cholesterol homeostasis, inflammatory processes, carcinogenesis, oxidative stress, and drug transport.

Quercetin administered alone had a considerable impact on the BFH12 cell transcriptome, significantly affecting the expression of more than one thousand genes. Similar results were obtained when treating BFH12 cells with curcumin [29]; conversely, resveratrol induced negligible transcriptional changes in the same cellular model [31]. Notably, of the natural extracts that we have so far tested in BFH12 cells, resveratrol was the most effective in mitigating AFB1-induced cytotoxicity. The present results seem to support the idea that the extent to which polyphenols affect BFH12 cells’ transcriptional profiles might not be positively correlated with their efficacy in mitigating AFB1 toxicity. In addition, a particularly interesting fact is that transcriptional profiles of cells exposed to QUE alone were nearly identical to those of cells co-treated with QUE and AFB1. This means that adding the mycotoxin had no substantial effect on cells previously “primed” with the flavonoid and that AFB1 did not produce transcriptional modifications additional to those triggered by QUE. However, further studies are needed to decipher the mechanisms underlying this peculiar transcriptional outcome.

#### 3.3.1. Cholesterol Metabolism

In the present study, we showed that QUE interferes with cholesterol metabolism, as previously demonstrated [56]. Indeed, in BFH12 cells, several genes involved in steroid metabolism were differentially regulated by QUE, such as SCARB1 (scavenger receptor class B type 1), which was also upregulated in this study. Conversely, this transcription factor has been reported to be downregulated by QUE in gut broilers, which in turn decreases triglycerides, total cholesterol, and low-density lipoproteins [57]. This result is also consistent with the observed upregulation of LDLR, responsible for LDL endocytosis and clearance, which has previously been reported to be regulated by QUE in rat models [58]. Other genes involved in steroid metabolism (e.g., FASN, HMGCR, and SCD) were upregulated by QUE, yet previous studies reported opposite results. For instance, several polyphenols, including QUE, have been recognized as FASN inhibitors [59,60]. In hepatocytes, HMGCR is a molecular target of many dietary polyphenols, being inhibited at both mRNA and enzymatic activity levels [61]. In hamsters fed with QUE diets, the mRNA of hepatic HMGCR was inhibited [62]; however, in the same study, HepG2 cells incubated with QUE did not show significant variations in HMGCR mRNA levels. By contrast, QUE was found to increase HMGCR activity in mice fed with a high-fat diet [63]. Likewise, HMGCR and its upstream and downstream genes, SREBF2 and LSS, were induced when cells exposed to QUE + AFB1 were compared to those exposed to AFB1 alone. These conflicting results are likely due to differences in cholesterol metabolism between species [64]. In this cell model, we might interpret this overexpression as a strategy of QUE to alleviate the AFB1-induced oxidative stress, as previously suggested in human lens epithelial cells subjected to UV-B [65].

#### 3.3.2. Inflammatory Processes

The data presented in this study showed that QUE’s mechanism of action might also encompass the regulation of the pathway of nuclear factor kappa-light-chain-enhancer of activated B cells (NF-kB), as previously suggested [66]. Notably, in the present study, the expression of this transcription factor, with well-recognized functions in regulating immune and inflammatory responses, was not directly modulated by QUE. Instead, a key gene that is normally inhibited via NF-kB deactivation, PTX3, was greatly downregulated by QUE. The importance of PTX3′s decrease in mediating QUE’s anti-inflammatory activity was previously demonstrated in human mesangial cells, in which it blocked the NF-kB signalling pathway and reduced renal damage [67]. Furthermore, suppression of NF-kB signalling is most likely linked to the downregulation of PTGS1 (i.e., COX1), an enzyme with critical roles in the pathophysiological progress of inflammation and cancer. Indeed, inhibitors or silencers of PTGS1 greatly attenuate the inflammatory response by negatively governing the NF-kB signalling pathway [68]. On the other hand, QUE induced the transcription of PTGS2 (i.e., COX2), which is usually activated in response to pro-inflammatory stimuli. In the literature, PTGS2 expression has been reported to be attenuated by QUE [69], so the induction observed here appears to be controversial. QUE’s anti-inflammatory properties were also confirmed by the potent downregulation of pro-inflammatory chemokines, in particular the CXCR3 ligands CXCL9, CXCL10, and CXCL11, which are important players in chronic liver diseases [70], inflammation [71], and cancer [72]. 

QUE’s potential to moderate inflammatory processes appeared to be crucial in mitigating AFB1-induced toxicity. Indeed, several genes with pro-inflammatory activity, such as interleukins and related receptors, chemokine ligands, and colony-stimulating factors, were downregulated in QUE + AFB1 vs. AFB1, as previously observed when pre-treating BFH12 with curcumin [29]. Interestingly, genes that mediate toxic pro-inflammatory responses (e.g., IL1A, IL6, and IL7R) are greatly induced by AFB1, both in vivo [73] and in vitro [74], including in BFH12 cells [26,32]. Importantly, the key protein that mediates the decrease in cytokine/chemokine release was, most likely, HMGB1, whose transcription was indeed lowered by QUE pre-treatment. This nuclear protein is involved in various liver injuries leading to inflammation and in regulating specific cell death responses, and QUE has previously been identified as its potential inhibitor [75]. For instance, in normal human hepatocytes, the HMGB1 transcript was lowered by QUE (from 25 to 100 µM), and the level of proteins playing a role in the corresponding signalling pathway was further reduced, while the production of ROS and the pattern of apoptosis were further suppressed [76]. 

Looking at the downregulated inflammatory mediators (e.g., CCL2, CCL20, CXCL5, and CSF3), we recognized the inhibition of the IL17-driven inflammation, whose hallmark is neutrophil accumulation [77]. Accordingly, the IL17 pathway was a significantly enriched KEGG pathway in the GSEA, together with the TNF and NF-kB pathways, which have several genes in common with the IL17 pathway and are all crucial in inflammatory responses.

Lastly, as far as QUE’s ability to hamper inflammatory processes is concerned, the downregulation by QUE of PDPN appeared to be of particular interest. This gene is normally upregulated during inflammation and cancer in different cell types. Some polyphenols, such as epigallocatechin-3-gallate and curcumin, have been reported to suppress PDPN expression in mouse tumours [78,79]. However, to our knowledge, the potential role of QUE as a PDPN inhibitor has never been reported before.

The central role of inflammation was also revealed by comparing genes regulated by AFB1 (compared to the control) and those regulated by QUE + AFB1 (compared to AFB1). Indeed, most of the shared DEGs showing opposite fold changes were related to inflammatory processes. This clearly demonstrated that in BFH12 cells, particularly under our experimental conditions, many benefits of QUE (here demonstrated in terms of reduced AFB1-induced cytotoxicity) are mediated by anti-inflammatory mechanisms.

#### 3.3.3. Oxidative Stress

A further highlight of the present study was QUE’s antioxidant potential, which is the reason why natural polyphenols are studied in the first place. Specifically, QUE alone increased the expression of MAFF, a small MAF transcription factor that dimerizes with nuclear factor erythroid 2–related factor 2 (NRF2), therefore activating antioxidant defences and resisting toxicant-induced oxidative stress [80]. It is well known that NRF2 pathway activation in response to cell stress leads to the downstream regulation of cytoprotective genes forming a network of cooperating enzymes involved in drug detoxification reactions (e.g., NQO1, CYPs, GSTs, and UGTs) and elimination (e.g., ABCB1 and BCRP) of pro-oxidants [81,82]. Additional NRF2-induced antioxidant enzymes are those mediating ROS elimination, such as enzymes producing and regenerating glutathione and redox cycling enzymes (e.g., thioredoxin, GPX, SOD, and CAT) [82]. In the present study, of the above-mentioned genes, QUE alone significantly impacted the expression of GPX1, GSTA3, GSTA2, and GSTM1. Although GPXs and GSTs are known targets of natural polyphenols [83], their upregulation, together with that of MAFF, seems not enough to suggest a strong activation of the NRF2 pathway in QUE-treated BFH12 cells. Accordingly, QUE did not increase NQO1 enzyme activity. Noteworthily, when comparing the QUE + AFB1 and AFB1 conditions, QUE’s antioxidant activity seems slightly more evident. Indeed, AFB1 greatly increased MDA production in BFH12 cells, but the pre-treatment with QUE was preventive. Accordingly, a previously published study reported that a QUE-supplemented diet significantly reduced the AFB1-induced increase in MDA in mouse brain tissue [84]. Importantly, the increase in MDA production is a general marker of oxidative stress, and the results obtained here demonstrate QUE’s antioxidant power in bovine hepatic cells. Regarding gene expression, GSTA2, GSTM1, and MGST1 were significantly upregulated by QUE pre-treatment. Notably, in BFH12, we observed that curcumin too induced the expression of these GSTs [29]. The most interesting regulated gene was NQO1, which was significantly induced by QUE pre-treatment. However, we showed that this transcriptional change was not reflected by an increased NQO1 catalytic activity. Taking these results together, we might speculate that in BFH12 cells, QUE induces a mild NRF2 activation by regulating the protein synthesis and/or activity of this key master regulator, rather than its mRNA transcription. This activation mostly results in an increased expression of antioxidant enzymes, mainly GSTs. Conversely, NQO1 does not seem to play a key role in the QUE-dependent antioxidant response to AFB1, as was demonstrated for curcumin and resveratrol [29,31]. Interestingly, compounds that decrease the inflammatory response by suppressing NF-kB signalling, as demonstrated for QUE, activate the NRF2 pathway [85]. Thus, NRF2 is an anti-inflammatory gene [86], and inhibition of inflammation by NRF2 is associated with the inhibition of the NF-kB pathway and pro-inflammatory cytokine production [87]. Although the molecular events regulating the interaction between NRF2 and inflammatory regulators remain largely unclear, phenolic antioxidants have been demonstrated to trigger these mechanisms [88].

#### 3.3.4. Carcinogenesis

Additional molecular targets of QUE in BFH12 cells were represented by genes involved in cancer progression. QUE alone largely downregulated some genes with a role in promoting cancer (e.g., OLFML2B, EMILIN2, and CD36), which is consistent with the anticancer potential of this natural extract [89]. In particular, QUE has already been shown to inhibit CD36 expression, thus protecting cells against cell proliferation, migration, and invasion [90,91]. Several genes involved in carcinogenesis were also modulated by QUE pre-treatment, as suggested by the GSEA suppression of the “MYC targets V1” and “MYC targets V2” hallmarks. MYC is a regulator of ribosomal biogenesis and protein synthesis [92]; its overexpression is suggested to activate several ribosomal proteins and enhance ribosomal biogenesis [93], which in turn affects cell proliferation. MYC upregulation occurs in up to 75% of cancers [94]. Some natural extracts, e.g., piperine and curcumin, have already been demonstrated to suppress the activity of MYC, resulting in anticancer effects [95,96]. In the present study, QUE did not directly suppress MYC, but it did suppress several genes whose expression is regulated by MYC. Among these were ribosomal proteins (e.g., SNRPD2, SNRPD3, RRP9, and RRP12), cell division cycle 20 (CDC20), heat shock protein family D (hsp60) member 1 (HSPD1), and bystin-like (BYSL). CDC20 has an oncogenic function in tumourigenesis [97], and its expression has been reported to be inhibited in vitro by curcumin and rottlerin, resulting in reduced survival of pancreatic cancer cells [98] and glioma cells [99]. HSPD1 encodes a mitochondrial chaperone promoting cell immortality and proliferation [100]; notably, its inhibition by curcumin has been associated with anti-glioma effects in vitro [101]. BYSL was reported to be crucial in hepatocarcinogenesis, both in vitro and in vivo. Moreover, inhibiting BYSL using RNA interference significantly decreased HCC cell proliferation in vitro, induced cell apoptosis, and partially arrested the cell cycle, leading to the failure of tumour formation [102].

#### 3.3.5. Transporters

AFB1 negatively affected the expression of several ABC transporters involved in the transport of fatty acids (e.g., ABCD1) and lipids (e.g., ABCA2, ABCA7, and ABCG1), whereas it induced the expression of xenobiotic efflux transporters, such as ABCB1 (also known as MDR1) and ABCC4 (MRP4). Intriguingly, the co-treatment with QUE and AFB1 reverted these transcriptional changes. ABCD1 is associated with peroxisomal β-oxidation, and in neuronal N2a cells, the 7-ketocholesterol-induced decrease in its mRNA and protein levels was counteracted by QUE, resveratrol, and apigenin [103]. ABCA2 is a key gene involved in maintaining the homeostasis of sterols, sphingolipids, and cholesterol, mainly in macrophages and neurons, but also in the liver [104]. ABCG1 is a porter of intracellular cholesterol, and ABCA7 is a bridge connecting cholesterol metabolism to the immune system and the body’s defence system [105]. Notably, ABCG1 has already been demonstrated in vitro (in macrophages) to be induced by QUE, thus protecting against ox-LDL-induced injury [106]. Looking at the xenobiotic efflux transporters, which are known to interact with several polyphenols [107], the role of ABCB1 in the transport of AFB1 is still unclear. Indeed, contrasting results were reported in vitro and in vivo [108,109], preventing us from interpreting the significance of ABCB1 downregulation observed here in the co-treatment with QUE and AFB1. For instance, curcumin ameliorates the duodenal toxicity of AFB1 in chicken by inducing the mRNA expression of ABCB1 and activating the encoded protein [110]. Likewise, a recent study has demonstrated that in the liver of broilers fed with QUE, ABCB1, mRNA and protein expression were upregulated [111]. On the other hand, QUE has been reported to negatively affect ABCB1 activity in HepG2 cells, thus potentiating the cytotoxic activity of anticancer drugs [112]. With regard to MRP4, it has been shown that QUE is most probably a substrate for this transporter, and its activity can be modulated by flavonoids [107]. To our knowledge, there is no evidence of a possible relationship between AFB1 and MRP4; however, the expression of ABCC4 is upregulated in HCC tissues. Based on this evidence, the ABCC4 downregulation observed in the present study after the co-treatment of QUE and AFB1 might mean that this molecular mechanism contributes to the overall mitigation of AFB1 toxicity in BFH12 cells [113].

## 4. Conclusions

To the best of our knowledge, QUE’s potential benefits in AFB1-exposed bovine cells have never been explored using an integrated approach combining cytotoxicity, RNA-seq, and post-transcriptional assays. The results of the present study, and those obtained in previous experiments on BFH12 cells, allow us to draw the following three conclusions. (1) QUE pre-treatment hampers AFB1-induced cytotoxicity, and this protective mechanism is mediated by the regulation of genes involved in lipid homeostasis, inflammatory and immune processes, and carcinogenesis. (2) In BFH12 cells, QUE possesses antioxidant activity that is mostly mediated by other pathways than previously identified for curcumin and resveratrol (e.g., diaphorase is not a major player). (3) QUE is a promising flavonoid, even though compared to curcuminoids and resveratrol, it exhibits a lower efficacy in mitigating aflatoxin-mediated toxicity in BFH12 cells. This does not mean that using QUE as a feed additive in cattle farming should be abandoned in favour of curcumin or resveratrol. Remarkably, there are plenty of additional factors to be considered when choosing a natural compound as a feed additive, such as the costs of the natural source of the compound (e.g., fruits and vegetables), extraction feasibility and costs, bioavailability, toxicity to tissues and organs, and overall molecular mechanisms (e.g., interference of the compound with physiological proteins, nutrients, or drug transporters), which might significantly impact the overall feasibility of a certain feeding strategy.

In conclusion, targeted molecular studies are envisaged to disentangle the role of specific pathways and genes in AFB1 toxicology and, consequently, to better characterize the protective role of QUE. In the meantime, in vivo studies implementing QUE-supplemented diets are recommended to assess QUE’s bioavailability and beneficial effects on aflatoxicosis.

## 5. Materials and Methods

### 5.1. Materials and Bovine Cell Line

Cell flasks and plates were purchased from Sarstedt (Verona, Italy). Williams’ E Medium, L-alanyl-l-glutamine, penicillin/streptomycin, and foetal bovine serum (FBS) were acquired from Biochrom (Biospa, Milan, Italy). AFB1, QUE (≥95% purity), and dimethyl sulfoxide (DMSO) were obtained from Sigma-Aldrich (St. Louis, MO, USA). PCB126 (99% purity) and AFM1 were purchased from Lab Service Analytica (Bologna, Italy), AFL from DBA Italia (Milano, Italy), and AFB1 13C17 from Orsell (Modena, Italy). All other chemicals used in the study were commercially available and of molecular biology grade. Solvents used for metabolite quantification were all of LC-MS grade.

The bovine SV40 large T-antigen-transduced foetal hepatocyte-derived cell line (BFH12) was kindly provided by Axel Schoeniger (Institute of Biochemistry, University of Leipzig, Germany) and cultured using the experimental procedures detailed in our previous paper [32]. For all the experiments, cells were used from passage 16 to passage 20.

### 5.2. Cytotoxicity

Cytotoxicity tests were conducted as previously reported [32]. Briefly, four days after seeding (6 × 10^3^ cells/well), BFH12 cells were exposed to increasing concentrations of QUE for a total of 64 h (16 + 48 h; range 5–250 μM). Cell viability was measured using WST-1 Cell Proliferation Reagent (Roche, Basel, Switzerland) and expressed as a percentage relative to the viability of cells exposed to the vehicle only (0.1% DMSO). Experiments were performed in triplicate, and each concentration was tested in sextuplicate.

Afterwards, we assessed the ability of QUE to reduce AFB1-induced cell mortality. Selected sub-cytotoxic concentrations (10, 20, and 30 μM) were defined based on the QUE dose–response curve and the resulting IC_50_ obtained in the present study. In agreement with the methodological approach adopted in our recent studies on AFB1 toxicity in the BFH12 cell line [29,31,32], we pre-treated cells with an aryl hydrocarbon receptor (AHR) agonist, i.e., the most potent dioxin-like PCB (PCB126). The rationale for this choice was the hypothesis that the metabolic competence of BFH12 cells could be lower than that of adult cells. Hence, PCB126 pre-treatment aimed at increasing cell responsiveness to AFB1.

### 5.3. Incubation of Cells for Gene Expression Analysis

**Figure 9:**
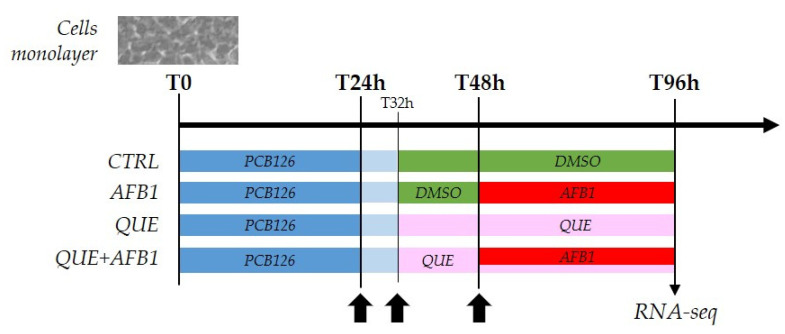
Cell treatments. Scheme reporting the cell treatments performed in this study and the resulting experimental groups assayed with the RNA-sequencing approach. DMSO = dimethyl sulfoxide. Arrows indicate complete medium changes (fresh medium and treatment solutions).

To assess the effects of AFB1 on the BFH12 transcriptional profile, cells were cultured in 6-well plates at a density of 5 × 10^4^ cells/well. Four days after seeding, monolayers were pre-treated with 1 nM PCB126 for 24 h. Then, cells were exposed to QUE (10, 20, or 30 μM), 3.6 μM AFB1, or their combination (Figure 9). Cells pre-treated with PCB126 and exposed to the vehicle (0.1% DMSO) were used as control. A total of four independent cell culture experiments were set up. All the details about the experimental procedures, including RNA isolation, RNA concentration, and quality assessment, are available in our previous papers [29,31,32].

### 5.4. Quantitative Real-Time PCR (qPCR)

Targeted qPCR analyses were preliminarily carried out to assess the effects of sub-cytotoxic QUE concentrations (i.e., 10, 20, and 30 μM). Target genes were those known to be somehow involved in AFB1 mechanistic toxicology (i.e., genes contributing to AFB1 biotransformation and/or involved in the antioxidant response) [32]. Four biological replicates (i.e., independent cell culture experiments) were performed. In summary, eight experimental conditions were taken into consideration: CTRL, QUE (10, 20, and 30 μM), AFB1, and QUE (10, 20, and 30 μM) + AFB1. The procedures of reverse transcription and qPCR amplification, primer sequences, and the principles of qPCR data analysis are described elsewhere [32].

### 5.5. Preparation of Libraries and RNA-seq

In addition to the CTRL and AFB1 experimental conditions, only cells exposed to the highest QUE concentration (i.e., 30 μM), either alone or in combination with AFB1, were subjected to RNA-seq. The QUE concentration of 30 μM was selected because it was the one provoking the most significant transcriptional variations using qPCR (Section 5.4). Three independent biological replicates per condition (i.e., independent cell culture experiments) were assessed. A total of 12 tagged RNA-seq libraries were prepared and sequenced following a 50-bp single-end strategy in an Illumina Hi-Seq 4000 instrument (Fasteris SA, Geneva, Switzerland). These libraries were prepared as detailed elsewhere [32], using Agilent’s SureSelect Strand Specific RNA Library Preparation Kit (Agilent Technologies, Santa Clara, CA, USA) and following the manufacturer’s instructions. Notably, the CTRL and AFB1 libraries were previously analysed in a stand-alone study assessing the transcriptional effects of PCB126 and AFB1 on BFH12 cells [32]. In the present study, these libraries were analysed again in the context of a larger dataset including new data (i.e., cells treated with QUE and QUE + AFB1).

### 5.6. Analysis of RNA-seq Data

Differential expression (DE) was conducted using edgeR [114] and grouping samples according to the treatment (i.e., CTRL, QUE, AFB1, and QUE + AFB1). Pairwise analyses were performed to assess the transcriptional changes induced by QUE, either alone (QUE vs. CTRL) or in combination with AFB1 (QUE + AFB1 vs. AFB1). Common and tagwise dispersions were estimated (*estimateDisp*), a linear model was fitted (*glmQLFit*), and the DEGs were determined using the function *glmTreat* with the following thresholds of significance: false discovery rate (FDR) ≤ 0.05 and log_2_ fold change (lfc) ≥ 1.

A functional interpretation of significant DEGs was obtained through GO and KEGG over-representation analysis (ORA) implemented in the R environment, using functions included in the ClusterProfiler package (i.e., *enrichGO*, *enrichKEGG*) [115]. Ensemble gene identifiers were used to establish two different gene lists (i.e., significantly up- and downregulated genes) and a “background” (i.e., all the expressed genes). Dot plots and gene-concept networks were also constructed via specific functions available in the ClusterProfiler package. Redundancy of gene ontology terms was removed using the *simplify* function (similarity cutoff = 0.5). Dot plots display the most significant enriched terms (*p* ≤ 0.05), while gene-concept networks highlight which genes were involved in the significant GO terms.

In order to analyse the transcriptional differences triggered by the cotreatment QUE + AFB1 (compared to AFB1 alone), a pre-ranked GSEA [116] was also performed. This statistical approach is helpful in determining whether gene sets that are defined a priori show statistically significant enrichment at either end of the ranking. A statistically significant enrichment value (Benjamini–Hochberg adjusted *p* ≤ 0.05) indicates that the biological activity (e.g., the biomolecular pathway), characterized by the gene set, is correlated with the supplied ranking. The ranked input was prepared as follows. A score (−log10 × *p* × sign(lfc)) was calculated from the edgeR differential expression results. A gene differentially expressed at a significant level (a low *p* close to 0) will be assigned a high score. The sign of lfc indicates whether the gene has an expression that is higher in the QUE + AFB1 condition (i.e., if lfc > 0, then the score will have a positive sign) or lower in the QUE + AFB1 condition (i.e., if lfc < 0, then the score will have a negative sign). In this way, all the expressed genes were ranked from top upregulated to top downregulated. The analysis was carried out using the *gseKEGG* and *GSEA* functions provided by the ClusterProfiler package [115]. While the first function was run for the enrichment of the KEGG pathways, the second one was run using the hallmark gene sets of the Molecular Signatures Database (MSigDB).

### 5.7. Analytical Investigations

Medium and cell samples were collected at the end of the experiment. Total AFB1, AFM1, and AFL were measured using LC-MS/MS in all the experimental conditions according to [32]. The LC-MS/MS system consisted of a Waters Acquity UPLC binary pump coupled to a Quattro Premier XE triple quadrupole mass spectrometer (Waters, Milford, MA, USA). Waters Acquity BEH C18 (50 × 2.1 mm, 1.7 µm) reversed-phase columns were used for the chromatographic separation. The instrument was equipped with an electrospray ionization source (ESI) operating in positive mode at a capillary voltage of 3.75 kV, with source and desolvation temperatures of 120 and 350 °C, respectively. The desolvation gas flow was 650 L/h, and the cone gas flow was 100 L/h. For each analyte, the following specific transitions were monitored: 313.1 > 284.7 *m*/*z* (CV 53 V; CE 25 eV) for AFB1, 329.1 > 272.7 *m*/*z* (CV 42 V; CE 26 eV) for AFM1, 297.1 > 268.4 *m*/*z* (CV 48 V; CE 20 eV) for AFL, and 330.3 > 300.6 *m*/*z* (CV 50 V; CE 22 eV) for the internal standard 13C17-AFB1.

### 5.8. Oxidative Stress

The oxidative damage induced by AFB1 and the possible counteracting effect of QUE were assessed by measuring the amount of MDA, a marker of lipid peroxidation. The experimental groups were the same as those used for the RNA-seq investigations. The MDA production was measured in six independent biological replicates (i.e., independent cell culture experiments) using the ab233471 lipid peroxidation colourimetric assay kit (Abcam, Prodotti Gianni S.p.A., Milan, Italy) according to [29].

### 5.9. Enzymatic Activity

As post-transcriptional confirmatory assays, CYP3A and NQO1 enzyme activity were measured. CYP3A and NQO1 are two key enzymes involved in AFB1 bioactivation and the antioxidant response, respectively. The former enzyme activity was measured using the P450-Glo^TM^ CYP3A4 assay, with luciferin-IPA as a substrate (Promega Corporation, Madison, WI, USA), and following the manufacturer’s instructions. For both enzymes, further protocol details are reported elsewhere [29]. As for NQO1, its catalytic activity was assessed using the ab184867 NQO1 activity assay kit (Abcam, Prodotti Gianni S.p.A., Milan, Italy).

### 5.10. Statistical Analysis

Dose–response curves were obtained using the GraphPad Prism software (version 8.0.2, San Diego, CA, USA), plotting a nonlinear regression [log(inhibitor) vs. normalized response, variable slope]. The IC_50_ and the goodness of fit (R squared) were obtained via the software.

The statistical analyses of cytotoxicity, qPCR data, and MDA content, as well as of NQO1 and CYP3A enzyme activities, were performed using a one-way ANOVA followed by Dunnett’s multiple comparisons test, with the level of significance set at *p* ≤ 0.05. The same statistical approach was adopted for the analytical data, but Tukey’s multiple comparisons test was used instead of Dunnett’s test.

## Data Availability

Raw Illumina sequencing data have been deposited in GenBank (SRA) under the BioProject accession PRJNA627332.

## Supplementary Materials

The following supporting information can be downloaded at https://www.mdpi.com/article/10.3390/toxins15090555/s1, Figure S1. Cytotoxicity; Figure S2. Effects of QUE in AFB1 biotransformation; Figure S3: MDS plot; Figure S4: MD plots; Figure S5: Over-representation analysis QUE vs. CTRL; Figure S6: Over-representation analysis: QUE+AFB1 vs. AFB1; Figure S7: Concordance between qPCR and RNA-seq results; Table S1: Sequencing and mapping results; Table S2: Differential expression analysis; Table S3: Enrichment analysis; Table S4: Over-representation analysis (GSEA).
