# Supplementary material for: Discovering the Protective Effects of Quercetin on Aflatoxin B1-Induced Toxicity in Bovine Foetal Hepatocyte-Derived Cells (BFH12)
Source: Toxins (Basel). 2023 Sep 6;15(9):555. doi: 10.3390/toxins15090555 (PMC10534839; doi:10.3390/toxins15090555)
Supplement: Supplementary file 1 [file toxins-15-00555-s001.zip › toxins-2539258.supp.pdf]

Supplementary material

Discovering the Protective Effects of Quercetin on Aflatoxin B1-Induced Toxicity in Bovine Foetal Hepatocyte-Derived Cells (BFH12)

Marianna Pauletto [1*†], Mery Giantin [1†], Roberta Tolosi [1], Irene Bassan [1‡], Anisa Bardhi [2], Andrea Barbarossa [2], Ludovica Montanucci [3], Anna Zaghini [2] and Mauro Dacasto [1]

[1] Department of Comparative Biomedicine and Food Science, University of Padua, Viale dell'Università 16, I-35020 Legnaro, Italy; mery.giantin@unipd.it (M.G.); roberta.tolosi@unipd.it (R.T.); irene.bassan2@gmail.com (I.B.); mauro.dacasto@unipd.it (M.D.)

[2] Department of Veterinary Medical Sciences, Alma Mater Studiorum—University of Bologna, Via Tolara di Sopra 50, Ozzano dell'Emilia, I-40064 Bologna, Italy; anisa.bardhi@unibo.it (A.B.); andrea.barbarossa@unibo.it (A.B.); anna.zaghini@unibo.it (A.Z.)

[3] Genomic Medicine Institute, Lerner Research Institute, Cleveland Clinic, 9500 Euclid Avenue, Cleveland, OH 44195, USA; montanl@ccf.org

* Correspondence: marianna.pauletto@unipd.it; Tel.: +39-049-827-2935

† These authors contributed equally to this work.

‡ Current address: Chelab srl—Mérieux NutriSciences Italia, Via Fratta 25, I-31023 Resana, Italy.

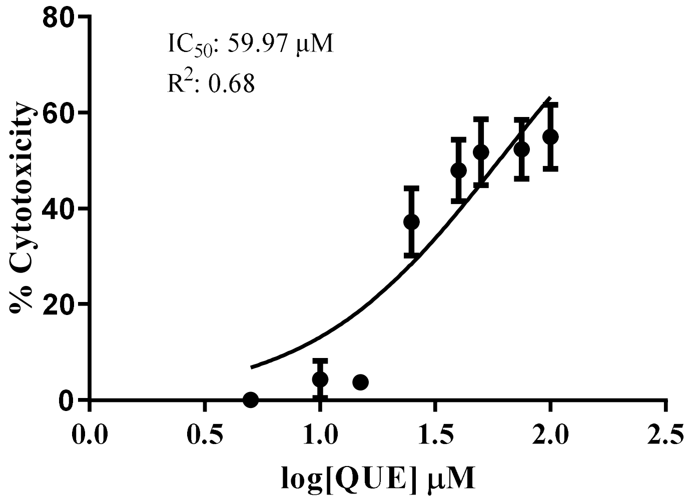

Figure S1. Cytotoxicity. Quercetin dose–response curve in BFH12 cells (64 h), based on three independent cell culture experiments, each one run in sextuplicate. Data are expressed in mean cytotoxicity rate ± standard error of the mean (SEM). IC_{50} and R^2 are also reported. QUE=quercetin

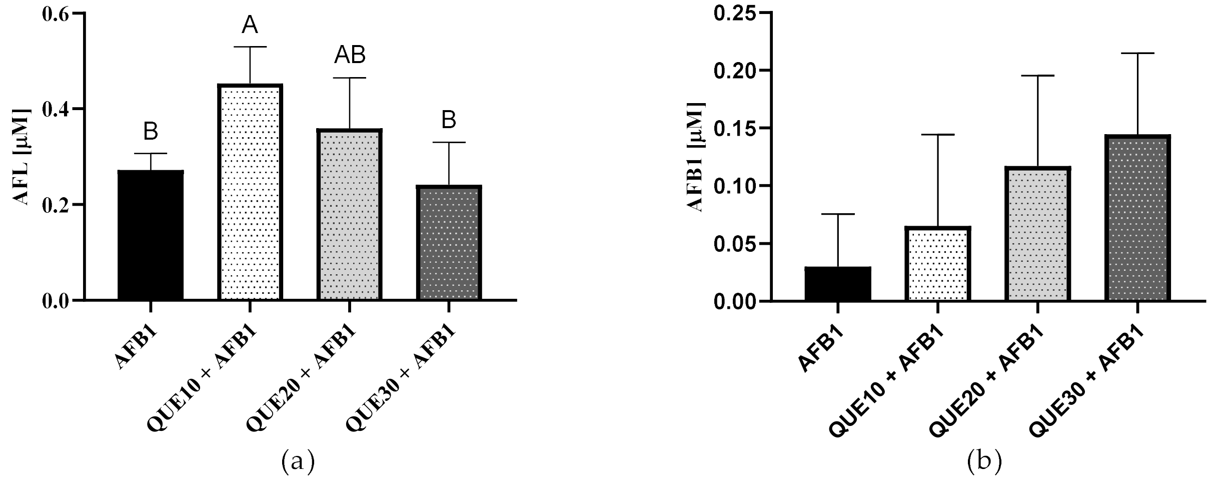

Figure S2. Effects of QUE in AFB1 biotransformation. Bars represent the amount (µM) of either AFL detected in the cellular medium (a) or AFB1 detected in the cellular pellet (b) after 48 h of exposure to 3.6 µM AFB1 alone or in combination with QUE increasing concentrations (10, 20, and 30 µM). Data are expressed as mean concentrations ± standard deviations of four independent cell culture experiments. Different letters above error bars indicate significant differences ($p \leq 0.05$) among groups (Tukey's post-hoc test). Graphs were obtained by means of GraphPad prism software. AFB1=aflatoxin B1; QUE=quercetin.

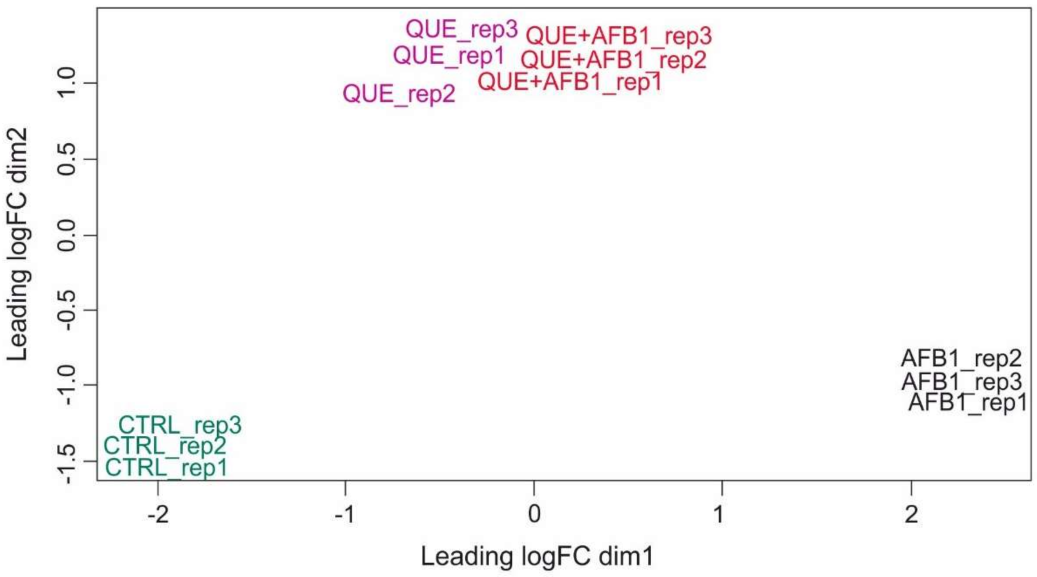

Figure S3. MDS plot. The plot shows distances between expression profiles of the twelve RNA-seq libraries evaluated in this study. Biological replicates are represented by rep1, rep2, and rep3 suffix. AFB1=aflatoxin B1; QUE=quercetin.

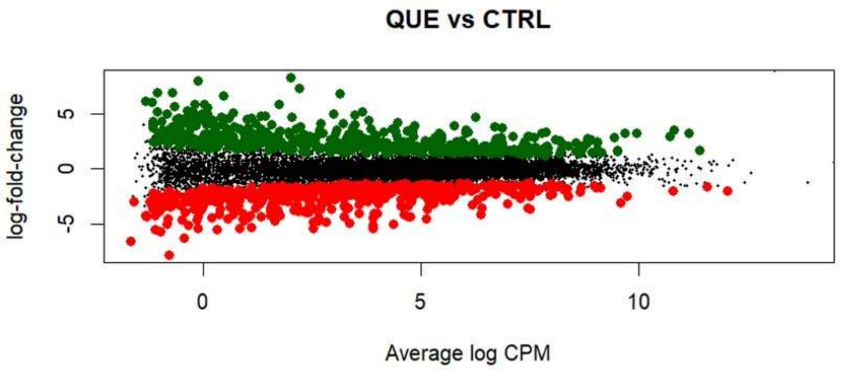

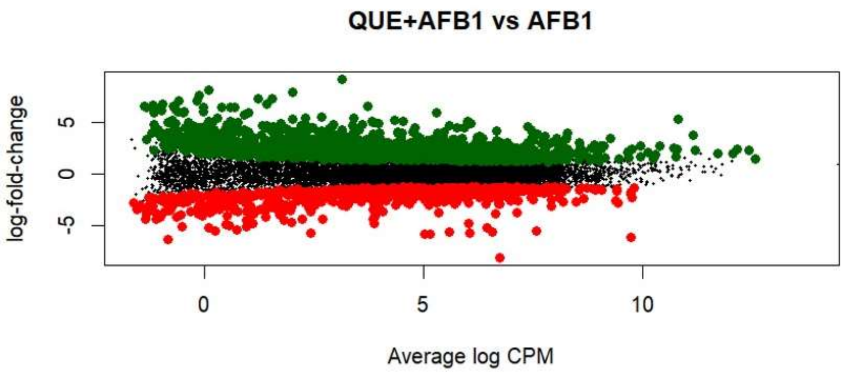

Figure S4. MD plots. Plots show the log-fold change and average abundance of each gene in the two comparisons ("QUE vs CTRL" and "QUE+AFB1 vs CTRL"). Genes with fold-changes significantly greater than 2 are highlighted in green (if up-regulated) or red (if down-regulated). CPM = Counts Per Million. AFB1=aflatoxin B1; QUE=quercetin.

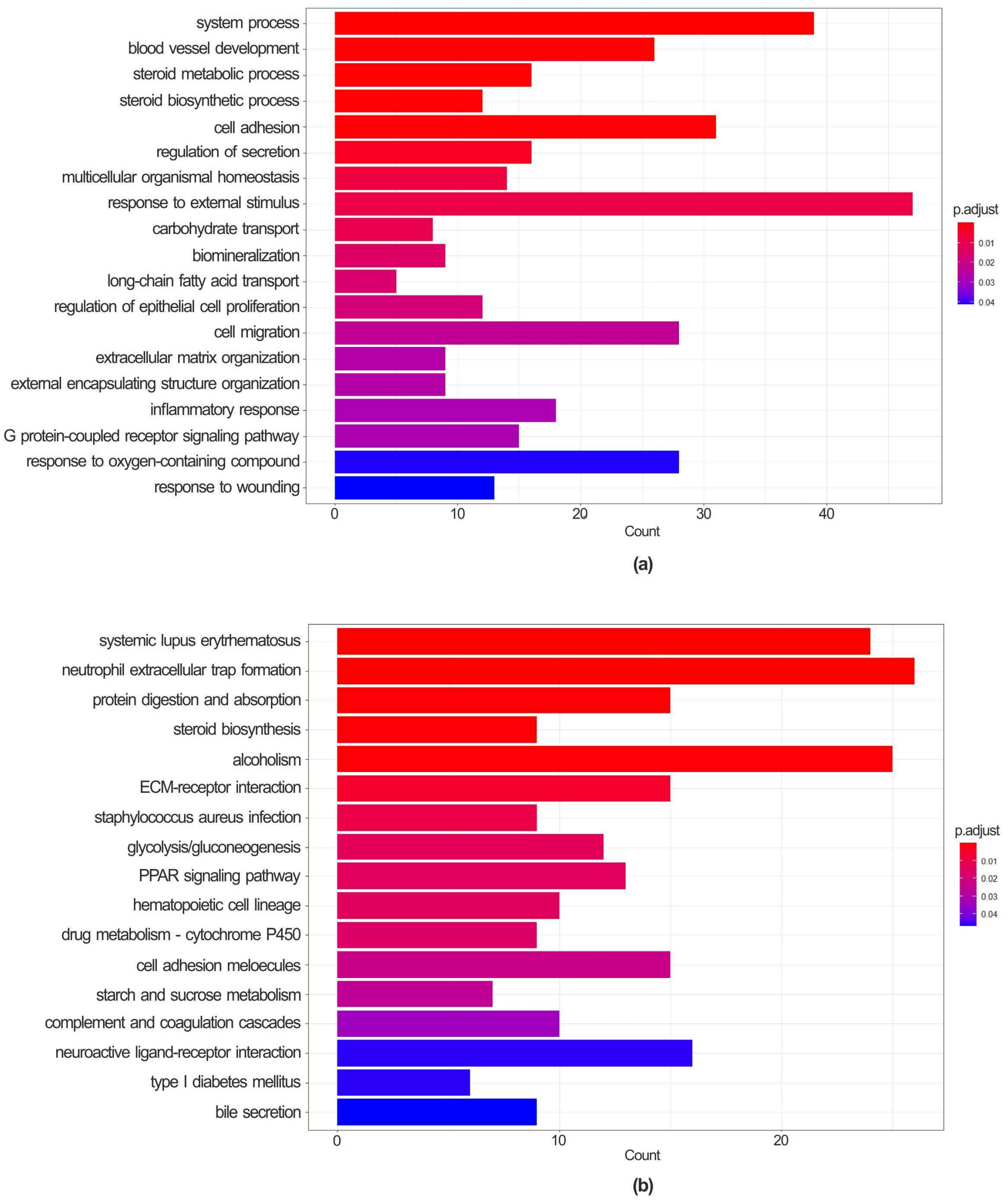

Figure S5. Over-representation analysis: QUE vs CTRL. Bar plots report the over-represented BP terms (a) and KEGG pathways (b) in the list of DEGs resulted from the pair-wise comparison "QUE vs CTRL". Count = number of DEGs in each enriched term/pathway. The color gradient reflects the significance level of each term. P-values were adjusted using the Benjamini-Hochberg method.

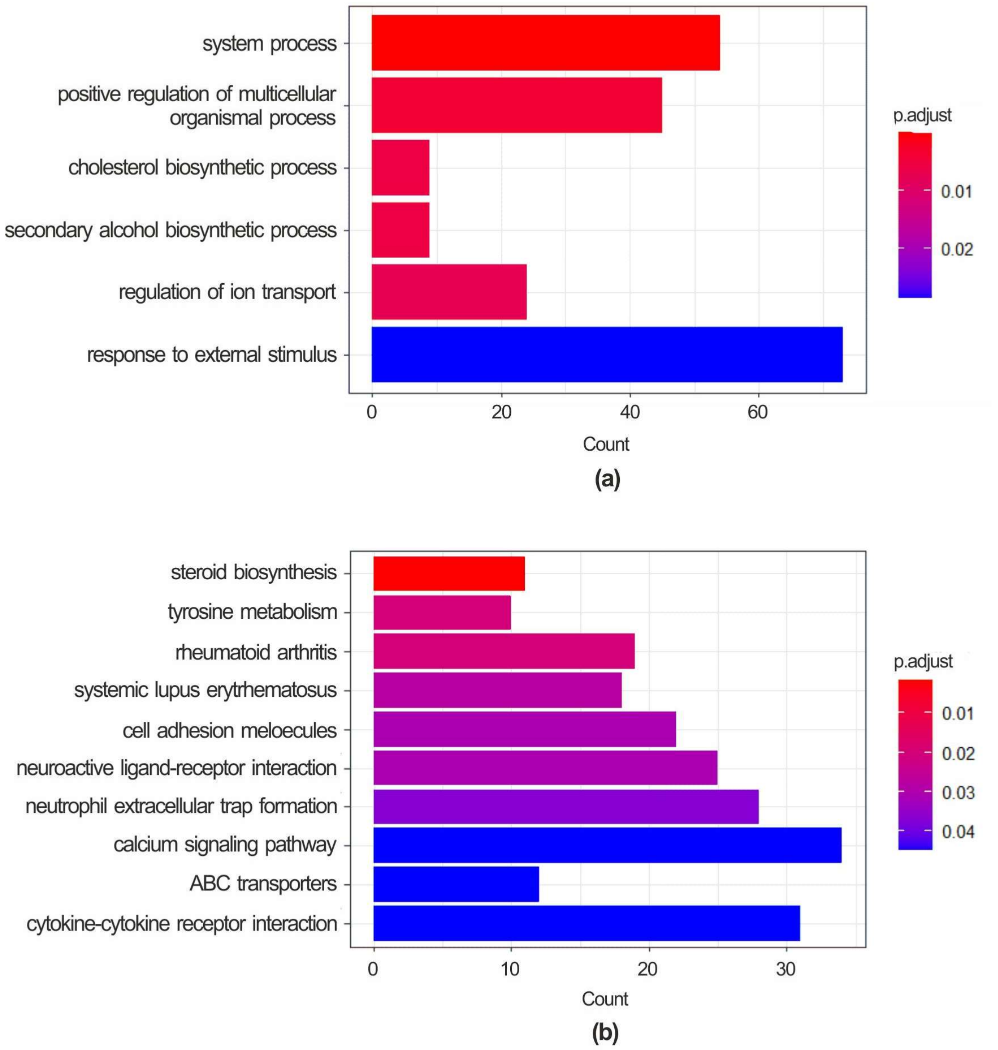

Figure S6. Over-representation analysis: QUE+AFB1 vs AFB1. Bar plots report the over-represented BP terms (a) and KEGG pathways (b) in the list of DEGs resulted from the pair-wise comparison "QUE+AFB1 vs AFB1". Count = number of DEGs in each enriched term/pathway. The color gradient reflects the significance level of each term. P-values were adjusted using the Benjamini-Hochberg method.

Gene	QUE + AFB1 vs AFB1
CYP1A1	↑*
CYP1B1	↑
CYP3A28	↓
GSTA1	ns
AHR	ns
AHRR	↑ / nd
ARNT	ns
NRF	ns
KEAP1	ns
CAT	ns
GPX1	ns
SOD1	↑ / ns
SOD2	↓
NQO1	↑

Figure S7. Concordance between qPCR and RNA-seq results. The figure reports the observed gene expression differences in the comparison QUE + AFB1 vs AFB1. Green and red colours highlight when the two methods agreed or disagreed, respectively. ns: not significant; ne: not detected; ↑: upregulated; ↓: downregulated; *by means of RNA-seq, this gene was induced with only marginal significance (i.e. FDR = 0.08).

Table S1. Sequencing and mapping results. The table reports the RNA-seq libraries sequenced, including for each of them: i) SRA accessions; ii) the number of raw reads obtained; iii) the number of reads after trimming and rRNAs removal; iv) the number of mapped reads (and the percentage of mapped reads).

SRA accession	Sample ID	Raw reads	Reads after trimming and rRNAs removal	Reads mapping (%)
SRR11586297	PCB126_B	22,928,709	22,823,199	22,628,132 (99.15)
SRR11586296	PCB126_C	25,807,296	25,653,873	25,436,649 (99.15)
SRR11586295	PCB126_D	43,005,307	42,793,203	42,432,959 (99.16)
SRR11586294	AFB1_B	20,399,647	20,286,542	20,106,412 (99.11)
SRR11586293	AFB1_C	23,877,815	23,632,276	23,428,800 (99.14)
SRR11586292	AFB1_D	10,774,628	10,714,111	10,614,084 (99.07)
SRR24958754	QUE_B	25,116,232	24,972,157	24,723,237 (99.00)
SRR24958753	QUE_C	22,334,139	22,173,375	21,991,767 (99.18)
SRR24958758	QUE_D	24,530,040	24,401,397	24,193,093 (99.15)
SRR24958757	QUE+AFB1_B	16,266,419	16,154,781	15,981,264 (98.193)
SRR24958756	QUE+AFB1_C	40,451,368	40,169,816	39,850,034 (99.20)
SRR24958755	QUE+AFB1_D	21,003,875	20,887,979	20,701,925 (99.11)

AFB1: aflatoxin B1; ID: identity; PCB: polychlorinated biphenyls; QUE: quercetin
